# Neuropsychiatric manifestations of Long COVID in India: a persistent problem 2.5 years after disease onset

**DOI:** 10.3389/fneur.2025.1704801

**Published:** 2025-11-12

**Authors:** Anurag Kumar Singh, Kamlesh Kumar, Mahaveer Singh, Tushar Jagawat, Deepak Nathiya, Balvir Singh Tomar, Savita Jagawat, Millenia Jimenez, Melissa Lopez, Janet Miller, Igor J. Koralnik

**Affiliations:** 1 Department of Pharmacy Practice, National Institute of Medical Sciences and Research, Jaipur, Rajasthan, India; 2 Department of Neurology, National Institute of Medical Sciences and Research, Jaipur, Rajasthan, India; 3 Department of Endocrinology, National Institute of Medical Sciences and Research, Jaipur, Rajasthan, India; 4 Department of Psychiatry, National Institute of Medical Sciences and Research, Jaipur, Rajasthan, India; 5 Institute of Pediatric Gastroenterology and Hepatology, National Institute of Medical Sciences and Research, Jaipur, Rajasthan, India; 6 Department of Psychology, National Institute of Medical Sciences and Research, Jaipur, Rajasthan, India; 7 Davee Department of Neurology, Feinberg School of Medicine, Northwestern University, Chicago, IL, United States; 8 Northwestern Medicine, Chicago, IL, United States; 9 Global Neurology Program, Havey Institute for Global Health, Northwestern University, Chicago, IL, United States

**Keywords:** Long COVID, post-acute sequelae of SARS-CoV-2 infection, PASC, neurology, India

## Abstract

**Background:**

Long COVID, also called post-acute sequelae of SARS-CoV-2 infection (PASC), is a multi-system syndrome affecting millions of people worldwide. Neuropsychiatric manifestations of Long COVID are particularly debilitating; however, they have not been reported in detail from India.

**Methods:**

This cross-sectional study compared the demographics, comorbidities, symptoms, and neuropsychiatric profiles of patients with Long COVID symptoms that began within 3 months of the initial SARS-CoV-2 infection and persisted for approximately 2.5 years at the time of evaluation. The study was conducted at NIMS University (Jaipur, Rajasthan, India).

**Results:**

Of 1,085 participants, 521 had COVID-19 pneumonia requiring hospitalization, while 564 had a mild initial respiratory presentation and were never hospitalized. On average 2.5 years after acute infection, our principal finding is that Long COVID differs based on initial disease severity. Post-hospitalization patients were older than the non-hospitalized group (43.24 vs. 36.15 years; *p* < 0.0001), had a higher body-mass index, and a more frequent prior history of lung disease (7.7% vs. 2.7%; *p* < 0.001). Overall, 69.6% of participants had at least one neurologic symptom, including myalgia (18.5%), dizziness (17.3%), headache (16.6%), pain (15.7%), anosmia (13.3%), brain fog (9.4%), numbness (6.0%), tinnitus (5.5%), and dysgeusia (3.7%), with only dizziness being more frequent in the post-hospitalization than the non-hospitalized group (20.3% vs. 14.5%; *p* = 0.012). Conversely, non-hospitalized patients had more frequent fatigue (23.2% vs. 6.0%; *p* < 0.0001), sleep problems (18.4% vs. 7.7%; *p* < 0.0001), chest pain (8.2% vs. 2.9%; *p* < 0.0001), and gastrointestinal symptoms (7.8% vs. 2.9%; *p* < 0.0001), while post-hospitalization patients more frequently complained of shortness of breath (9.6% vs. 2.7%; *p* < 0.0001). Overall, 4.9% of participants had cognitive dysfunction on the MMSE test, while 10.4% suffered from stress, and 12.7 and 9.2% had anxiety and depression symptoms, respectively, on DASS assessment, without significant differences between the two groups.

**Conclusion:**

Our study highlights differences in demographics and clinical presentation of Long COVID between post-hospitalization and non-hospitalized individuals in India. The high frequency of neurologic manifestations 2.5 years after disease onset underscores the need for early detection and targeted interventions.

## Introduction

The COVID-19 pandemic has had a profound global impact, with over 779 million confirmed cases and 7.1 million deaths reported worldwide, according to the World Health Organization (WHO) (1). In India there have been 45.1 million confirmed cases of COVID-19 with 534,000 reported deaths (2). Infection with the SARS-CoV-2 virus extends beyond its acute phase, with post-acute sequelae of SARS-CoV-2 infection (PASC), also called Long COVID, emerging as a major public health challenge in India. Persistent symptoms affecting the respiratory, cardiovascular, musculoskeletal, and central nervous systems can last for weeks or months, placing a significant burden on individuals and healthcare systems (3, 4). Research from North India reports a high prevalence of Long COVID, with nearly 40% of recovered individuals experiencing symptoms like fatigue, dyspnea, myalgia, and cognitive dysfunction beyond 12 weeks (5). Similar findings were observed in Haryana, where individuals who had previously tested positive for COVID-19 exhibited higher rates of lingering symptoms, particularly persistent cough, joint pain, and fatigue (6).

In Kerala, a study on patients with mild or asymptomatic infections found that even those with minimal symptoms during the acute phase were susceptible to prolonged manifestations, including fatigue, joint pain, and breathlessness, indicating that Long COVID is not limited to those with severe initial illness (7). Emerging evidence suggests that thyroid dysfunctions are relatively common in individuals with Long COVID and may contribute to persistent fatigue, cognitive impairment, and other neurologic manifestations (8). The burden of Long COVID is further compounded by multisystem inflammatory syndrome in adults (MIS-A), which has been reported in some post-COVID cases and is associated with cardiac dysfunction, gastrointestinal disturbances, and systemic inflammation (9). The varied nature of post-COVID symptoms necessitates a structured and multidisciplinary approach to management, incorporating targeted follow-ups, community outreach programs, and telemedicine services (6).

Long COVID has emerged as a major healthcare concern requiring urgent attention. The decentralized Data4Life study in India has demonstrated the potential of digital health technologies in capturing real-world data on post-COVID sequelae, enabling researchers to better understand the long-term consequences and formulate evidence-based interventions (10). Additionally, predictive risk models have been developed to identify individuals at higher risk of developing prolonged symptoms, allowing for early interventions and better patient outcomes (4). Despite the growing body of literature, there remains a need for standardized definitions and diagnostic criteria to improve the classification and management of Long COVID across diverse populations.

As the world transitions beyond the acute phase of the COVID-19 pandemic, the long-term health consequences of the virus continue to pose challenges, and a significant proportion of individuals continue to experience lingering symptoms of Long COVID that affect their daily lives (3).

Neurologic manifestations of PASC, (Neuro-PASC) are the main cause of visits to post-COVID clinics (11) and are among be the most debilitating (12, 13). However, their incidence in the Indian population has not been studied in detail. This comprehensive analysis aims to explore the long-term implications of neurocognitive and psychiatric manifestations in Long-COVID patients. Since Neuro-PASC manifestations differ based on COVID-19 severity (14), we analysed post-hospitalization and non-hospitalized patients separately. By synthesizing current evidence, we seek to provide insights into effective diagnostic strategies, therapeutic interventions, and the broader impact of Long-COVID on mental well-being and quality of life in the Indian population.

## Materials and methods

This prospective observational study was conducted at the Department of Neurology of NIMS University Rajasthan, Jaipur, India in collaboration with Northwestern University Feinberg School of Medicine, Chicago, USA. Data was extracted from the NIMS Electronic Medical Record database for the timeframe of March 1st, 2020 to March 31st, 2023. A total of 18,799 confirmed COVID-19-positive cases were identified, including 11,807 hospitalized patients and 6,992 non-hospitalized patients. We contacted 6,992 non-hospitalized individuals, out of which 564 responded, and 6,992 hospitalized individuals, out of which 521 responded. In total, 1,085 patients were assessed.

Patients were evaluated either in person or telephonically. Among the post-hospitalization group (*n* = 521), 491 were assessed in person and 30 by phone. Among the non-hospitalized group (*n* = 564), 278 patients were assessed in person and 286 by phone.

### Variables and measurements

The study analysed demographic, clinical, neurocognitive, and psychiatric variables. Demographic data included age, gender, education, occupation, marital status, area of living, smoking and alcohol consumption status. Neurocognitive function was assessed using the Mini-Mental State Examination (MMSE), and psychiatric symptoms were evaluated using the Depression Anxiety Stress Scales (DASS-21).

### Mini-Mental State Examination (MMSE)

The MMSE is a 30-point cognitive screening tool used to assess orientation, memory, attention, language, and visuospatial abilities. It is widely used in clinical and research settings to detect cognitive impairment and monitor changes over time. A score of 24 or higher is generally considered normal, while lower scores indicate varying degrees of cognitive dysfunction. The MMSE was administered either in-person or via telephone (15).

### Depression Anxiety Stress Scales (DASS-21)

The DASS-21 is a 21-item self-report questionnaire designed to assess psychological distress across three subscales: depression, anxiety, and stress. Each item is scored on a four-point Likert scale, and total scores are derived for each subscale. The DASS-21 is commonly used in research and clinical practice to evaluate emotional well-being and mental health status. The DASS-21 was administered either in-person or via telephone (16).

### Study groups

Participants were categorized into two groups based on hospitalization status during the acute phase of COVID-19. The post-hospitalization group included 521 patients who required hospitalization due to COVID-19 and subsequently developed persistent neurological and psychiatric symptoms. The non-hospitalized group comprised 564 patients who did not require hospitalization but still experienced post-COVID neurocognitive and psychiatric symptoms.

### Statistical analysis

Data were analysed using SPSS v28. Continuous variables were assessed for normality using the Shapiro–Wilk test and visual inspection of histograms and Q-Q plots. Normally distributed variables (e.g., age, height, weight) were expressed as mean ± standard deviation (SD) and compared between groups using the independent samples *t*-test. Non-normally distributed variables (e.g., months since symptom onset, MMSE scores, DASS-21 subscale scores) were expressed as median with interquartile range (IQR) and compared using the Mann–Whitney U test. Categorical variables (e.g., gender, education, comorbidities, symptom prevalence) were summarized as counts and percentages and compared between post-hospitalized and non-hospitalized groups using the Chi-square test or Fisher’s exact test where expected cell counts were <5. All statistical tests were two-tailed, and a *p*-value < 0.05 was considered statistically significant.

### Ethical considerations

**Figure 1:**
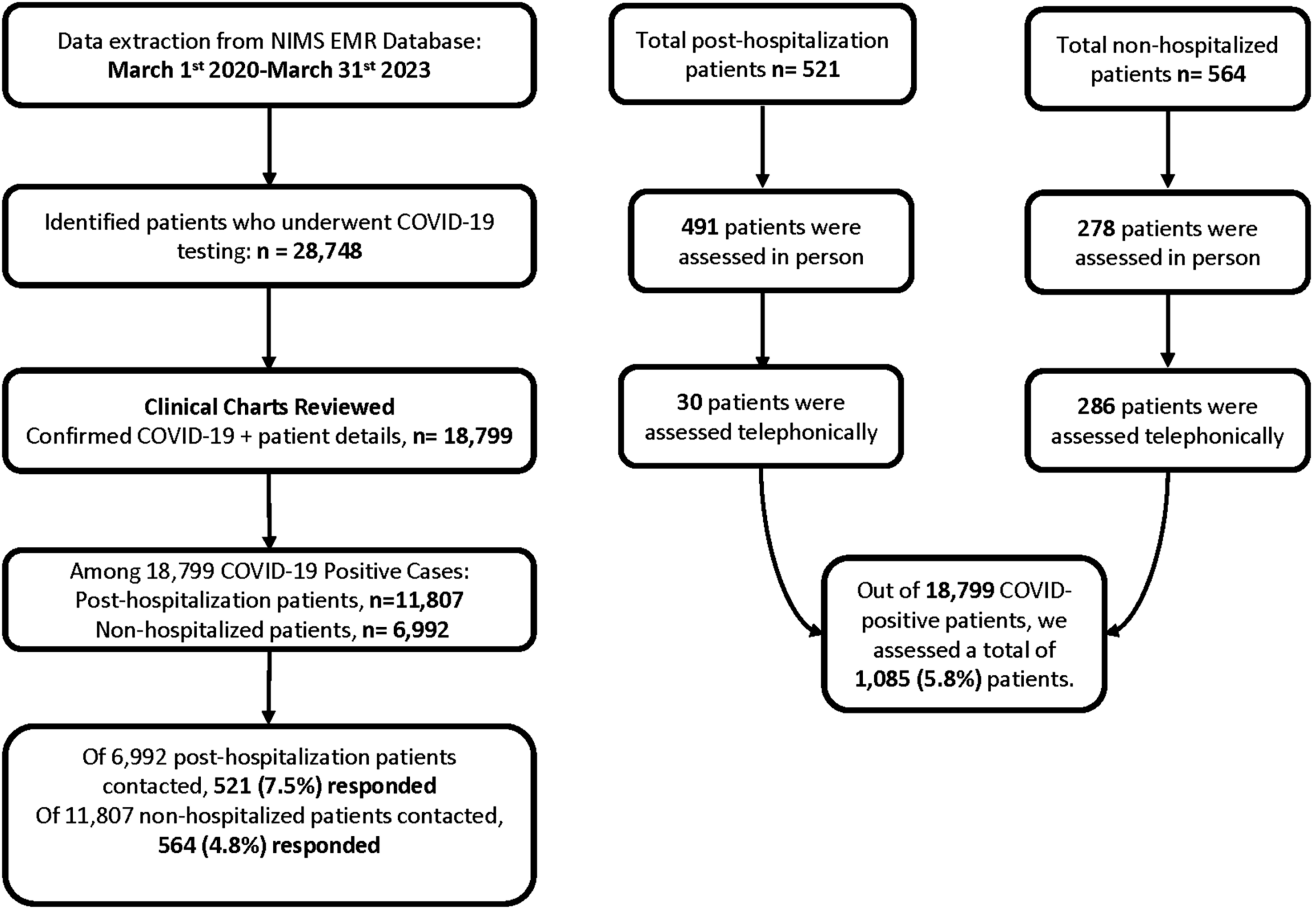
Workflow of participants recruitment.

The current study follows the STROBE statement for observational studies (17). The study protocol was approved by the Institutional Ethics Committee (IEC) of NIMS University Rajasthan, Jaipur (IEC/P-836/2024). Additionally, ethical clearance was obtained from Northwestern University, with IRB ID STU00221890. Written informed consent was obtained from all participants per the Declaration of Helsinki and national regulations for human health research (Figure 1).

## Results

### Demographic and clinical characteristics

Table 1 shows the demographic and clinical characteristics of 1,085 participants who were post-hospitalization (*n* = 521) and non-hospitalized (*n* = 564) for COVID-19. The median time since symptom onset was similar across groups (28 vs. 31 months; *p* = 0.911). Both groups were comparable in gender distribution, with a higher frequency of males, representing 57.2% in post-hospitalization and 56.0% in non-hospitalized groups. Patients predominantly resided in rural areas (64.8%). COVID-19 vaccination rate overall was 87.6%, though significantly higher among non-hospitalized compared to post-hospitalization patients (90.1% vs. 84.8%, *p* < 0.0001). Education distribution showed no significant differences between groups with graduates constituting the largest subgroup (36.2% overall). BMI categories revealed notable differences, with obesity significantly more frequent among post-hospitalization compared to non-hospitalized patients (33.6 vs 13.5%; *p* < 0.0001). The mean body weight was also significantly greater in the post-hospitalization group compared to non-hospitalized (71.74 ± 16.21 vs 68.51 ± 11.14 kg; *p* < 0.0001). Smoking, alcohol, and tobacco usage patterns did not differ significantly between groups. The mean height, gender distribution, education level, family type, religion, and employment status showed no statistically significant differences between the two groups.

**Table 1:** Study subjects’ demographics in post-hospitalization and non-hospitalized patients.

Variables	Overall (1085)	Post-hospitalization (521)	Non-hospitalized (564)	*p*-value
Age (years) Mean (SD)	39.56 (13.34)	43.24 (10.89)	36.15 (14.93)	**<0.0001**
Months from symptoms onset median (IQR)	30 (28–31)	28 (26–30)	31 (29–33)	0.911
Gender				
Male, *n* (%)	614 (56.6)	298 (57.2)	316 (56.0)	0.713
Female, *n* (%)	471 (43.4)	223 (42.8)	248 (44.0)	
Area of living				
Rural, *n* (%)	703 (64.8)	337 (64.7)	366 (64.9)	0.949
Urban, *n* (%)	382 (35.2)	184 (35.3)	198 (35.1)	
Religion				
Hindu, *n* (%)	998 (91.9)	479 (92.0)	519 (91.8)	0.955
Muslim, *n* (%)	80 (7.4)	39 (7.5)	41 (7.3)	
Christian, *n* (%)	7 (0.6)	3 (0.6)	4 (0.7)	
Vaccination				
Yes, *n* (%)	950 (87.6)	442 (84.8)	508 (90.1)	**<0.0001**
No, *n* (%)	135 (12.4)	79 (15.2)	56 (9.9)	
Family type				
Nuclear, *n* (%)	475 (43.8)	225 (43.2)	250 (44.3)	0.778
Joint, *n* (%)	553 (51.0)	269 (51.6)	284 (50.4)	
Extended, *n* (%)	57 (5.3)	27 (5.2)	30 (5.3)	
Education				
Illiterate, *n* (%)	296 (27.3)	142 (27.3)	154 (27.3)	0.92
High school, *n* (%)	232 (21.4)	110 (21.1)	122 (21.6)	
Intermediate, *n* (%)	116 (10.7)	56 (10.8)	60 (10.6)	
Graduate, *n* (%)	393 (36.2)	190 (36.5)	203 (36.0)	
Professional degree, *n* (%)	48 (4.4)	23 (4.4)	25 (4.4)	
Occupation				
Employed, *n* (%)	531 (49.0)	258 (49.5)	273 (48.4)	0.716
Unemployed, *n* (%)	554 (51.0)	263 (50.5)	291 (51.6)	
Height (cm) Mean (SD)	168.61 (10.4)	168.23 (10.25)	168.99 (10.54)	0.226
Weight (kg) Mean (SD)	70.13 (14.09)	71.74 (16.21)	68.51 (11.14)	**<0.0001**
BMI categories				
Underweight, *n* (%)	174 (16.0)	94 (18.0)	80 (14.2)	**<0.0001**
Normal, *n* (%)	387 (35.7)	142 (27.2)	245 (43.4)	
Overweight, *n* (%)	273 (25.2)	110 (21.1)	163 (28.9)	
Obese, *n* (%)	251 (23.1)	175 (33.6)	76 (13.5)	
Smoking history				
Yes, *n* (%)	574 (52.9)	278 (53.4)	296 (52.5)	0.808
No, *n* (%)	511 (47.1)	243 (46.6)	268 (47.5)	
Current alcohol use				
Yes, *n* (%)	375 (34.6)	181 (34.8)	194 (34.4)	0.949
No, *n* (%)	710 (65.4)	340 (65.2)	370 (65.6)	
Current tobacco use				
Yes, *n* (%)	393 (36.2)	190 (36.5)	203 (36.0)	0.899
No, *n* (%)	692 (63.8)	331 (63.5)	361 (64.0)	

*P*-values that are significant *p* < 0.05 are highlighted in bold.

### Prevalence of comorbidities associated with COVID-19

Table 2 presents the prevalence of comorbidities among post-hospitalized and non-hospitalized study participants. Hypertension was observed in 10.8% of post-hospitalized and 11.2% of non-hospitalized individuals, showing no significant difference (*p* = 0.824). Similarly, diabetes mellitus was reported in 9.6% of post-hospitalized and 9.0% of non-hospitalized participants (*p* = 0.754), while dyslipidemia showed nearly identical prevalence in both groups (8.1% vs. 8.0%; *p* = 0.960). Notably, lung disease (COPD/asthma) was significantly more prevalent among post-hospitalized patients (7.7%) compared to their non-hospitalized counterparts (2.7%; *p* < 0.001). Other less common comorbidities, including those grouped as “others” (1.5% vs. 1.8%; *p* = 0.784). The markedly higher rate of lung disease in post-hospitalized individuals underscores a possible association between pre-existing respiratory conditions and the need for hospitalization.

**Table 2:** Study subjects’ comorbidities in post-hospitalization and non-hospitalized patients.

Comorbidity, *n* (%)	Overall (*n* = 1,085)	Post-hospitalization (*n* = 521)	Non-hospitalized (*n* = 564)	*p*-value
Hypertension (HTN)	119 (11.0)	56 (10.8)	63 (11.2)	0.824
Diabetes mellitus	101 (9.3)	50 (9.6)	51 (9.0)	0.754
Dyslipidaemia	87 (8.0)	42 (8.1)	45 (8.0)	0.960
Lung disease (COPD/Asthma)	55 (5.1)	40 (7.7)	15 (2.7)	**<0.001**
Others	18 (1.7)	8 (1.5)	10 (1.8)	0.784
Hypothyroidism	15 (1.4)	10 (1.9)	5 (0.9)	0.194
Rheumatoid arthritis	11 (1.0)	5 (1.0)	6 (1.1)	0.864
Cardio-vascular disease	10 (0.9)	6 (1.2)	4 (0.7)	0.534
Crohn’s disease	8 (0.7)	5 (1.0)	3 (0.5)	0.491

*P*-values that are significant *p* < 0.05 are highlighted in bold.

### Neurologic symptoms post-COVID-19

Table 3 provides an overview of neurologic symptoms among post-hospitalization and non-hospitalization COVID-19 patients. Of all patients, 69.6% experienced at least one neurologic symptom, without significant difference between the two groups.

**Table 3:** Study subjects’ neurologic symptoms and signs in post-hospitalization and non-hospitalized patients.

Neurologic symptoms, *n* (%)	Overall (*n* = 1,085)	Post-hospitalization (*n* = 521)	Non-hospitalized (*n* = 564)	*p*-value
At least 1 symptom	755 (69.6)	330 (63.3)	425 (75.4)	0.068
No of neurologic symptoms (median [IQR])				
Myalgia	201 (18.5)	103 (19.8)	98 (17.4)	0.311
Dizziness	188 (17.3)	106 (20.3)	82 (14.5)	**0.012**
Headache	180 (16.6)	92 (17.6)	88 (15.6)	0.363
Pain	170 (15.7)	80 (15.4)	90 (16.0)	0.775
Anosmia	144 (13.3)	76 (14.6)	68 (12.1)	0.22
Brain Fog	102 (9.4)	40 (7.7)	62 (11.0)	0.062
Numbness	65 (6.0)	28 (5.4)	37 (6.6)	0.407
Tinnitus	60 (5.5)	36 (6.9)	24 (4.3)	0.056
Dysgeusia	40 (3.7)	24 (4.6)	16 (2.8)	0.122
Neurologic signs, *n* (%)^*^	769	491	278	
At least 1 sign	83 (10.8)	33 (6.7)	50 (17.9)	**<0.0001**
Sensory dysfunction	33 (4.3)	21 (4.3)	12 (4.3)	0.979
Gait dysfunction	30 (3.9)	22 (4.5)	8 (2.9)	0.270
Motor dysfunction	30 (3.9)	24 (4.9)	6 (2.2)	0.06
Short-term memory loss	30 (3.9)	10 (2)	20 (7.2)	**0.0004**
Blurry vision	17 (2.2)	12 (2.4)	5 (1.8)	0.558
Movement disorders	8 (1)	4 (0.8)	4 (1.4)	0.469
Major cognitive issues	6 (0.8)	4 (0.8)	2 (0.7)	1

* Neurologic signs were calculated only from participants evaluated in-person.

*P*-values that are significant *p* < 0.05 are highlighted in bold.

Dizziness emerged as a significantly more frequent symptom in the hospitalization group (20.3%) compared to the non-hospitalization group (14.5%; *p* = 0.012). Other neurologic symptoms like myalgia (19.8% vs. 17.4%), headache (17.6% vs. 15.6%), pain (15.4% vs. 16.0%), and anosmia (14.6% vs. 12.1%) did not show statistically significant differences between the groups. Notably, brain fog (7.7% vs. 11.0%; *p* = 0.062), tinnitus (6.9% vs. 4.3%; *p* = 0.056), dysgeusia (4.6% vs. 2.8%; *p* = 0.122), and blurry vision (2.3% vs. 0.9%; *p* = 0.06) demonstrated trends toward significance, suggesting a higher occurrence of certain sensory-related symptoms among those who had been hospitalized. Symptoms such as numbness (5.4% vs. 6.6%) appeared comparably in both groups without significant differences.

Compared to neurologic symptoms, abnormal neurologic findings measured only in participants evaluated in-person were relatively rare. Only 10.8% of these patients had at least one abnormal neurologic sign on exam, which was more frequent among non-hospitalized patients (6.7% vs. 17.9%; *p* < 0.0001), who also harbored more frequent short term memory loss (2% vs. 7.2%; *p* < 0.0004). Conversely, there was trend for motor dysfunction (4.9% vs. 2.2%; *p* < 0.06) to be more prevalent in the hospitalization group, reflecting possible neuromuscular complications associated with severe illness and prolonged hospitalization. Other signs were rare and not significantly different between the groups.

### General clinical symptoms post-COVID-19

Table 4 presents a comparative overview of general symptoms experienced by post-hospitalization and non-hospitalization COVID-19 patients. Fatigue was significantly more prevalent among the non-hospitalization group (23.2%) compared to the hospitalization group (6.0%; *p* < 0.0001), as were sleep problems (18.4% vs. 7.7%; *p* < 0.0001), suggesting a higher burden of general post-COVID symptoms in individuals who were not hospitalized. Conversely, shortness of breath was significantly more common in the hospitalization group (9.6%) than in the non-hospitalization group (2.7%; *p* < 0.0001), likely indicating lingering respiratory sequelae following hospitalization. Chest pain (8.2% vs. 2.9%; *p* < 0.0001) and gastrointestinal symptoms (7.8% vs. 2.9%; *p* < 0.0001) were also significantly more frequent among the non-hospitalization group. Anxiety (12.8% vs. 10.7%; *p* = 0.303) and depression (8.7% vs. 9.8%; *p* = 0.531) showed no statistically significant differences between the two groups.

**Table 4:** Prevalence of general symptoms in post-hospitalized and non-hospitalized patients.

General symptoms, *n* (%)	Overall (*n* = 1,085)	Post-hospitalization (*n* = 521)	Non-hospitalized (*n* = 564)	*p*-value
Fatigue	162 (14.9)	31 (6.0)	131 (23.2)	**<0.0001**
Sleep problems	144 (13.3)	40 (7.7)	104 (18.4)	**<0.0001**
Anxiety	128 (11.8)	56 (10.7)	72 (12.8)	0.303
Depression	100 (9.2)	51 (9.8)	49 (8.7)	0.531
Shortness of breath	65 (6.0)	50 (9.6)	15 (2.7)	**<0.0001**
Chest pain	61 (5.6)	15 (2.9)	46 (8.2)	**<0.0001**
GI symptoms	59 (5.4)	15 (2.9)	44 (7.8)	**<0.0001**

*P*-values that are significant *p* < 0.05 are highlighted in bold.

### Assessment of cognitive function using Mini-Mental State Examination (MMSE scores)

Table 5 summarizes the cognitive function assessment using the Mini-Mental State Examination (MMSE) among post-hospitalized and non-hospitalized COVID-19 patients. Overall, the majority of patients 95.1% showed no cognitive impairment with no statistically significant difference between groups. Mild cognitive impairment was similarly observed in both groups 4.4% post-hospitalization vs. 4.3% non-hospitalized. Dementia was rarely detected, appearing in only 0.4% of post-hospitalized and 0.7% of non-hospitalized participants. These findings suggest that cognitive outcomes post-COVID-19 infection were broadly similar irrespective of hospitalization status, with minimal prevalence of cognitive deficits overall.

**Table 5:** Cognitive function assessment (MMSE) among post-hospitalized and non-hospitalized patients.

Cognitive Function (MMSE), *n* (%)	Response	Overall (*n* = 1,084)	Post-hospitalization (*n* = 521)	Non-hospitalized (*n* = 563)	*p*-value
Cognitive function	No cognitive impairment	1,031 (95.1)	496 (95.2)	535 (95.0)	0.765
	Mild cognitive impairment	47 (4.3)	23 (4.4)	24 (4.3)	
	Dementia	6 (0.6)	2 (0.4)	4 (0.7)	

### Psychological assessment of stress, anxiety, and depression (DASS-21 scores)

Table 6 illustrates the distribution of stress, anxiety, and depression levels among post-hospitalization and non-hospitalized COVID-19 patients. Overall, the majority of patients (89.6%) exhibited normal stress levels, anxiety (87.3%) and depression scores (90.8%) without statistically significant differences between groups. These results indicate generally low psychological distress across groups, with no meaningful variation in stress, anxiety, and depression based on hospitalization status.

**Table 6:** Distribution of stress, anxiety, and depression levels (DASS) among post-hospitalization and non-hospitalized patients.

DASS category, *n* (%)	Response	Overall (*n* = 1,084)	Post-hospitalization (*n* = 521)	Non-hospitalized (*n* = 563)	*p*-value
Stress (DASS)	Normal	971 (89.6)	476 (91.4)	495 (87.9)	0.288
	Mild	77 (7.1)	30 (5.8)	47 (8.3)	
	Moderate	33 (3.0)	13 (2.5)	20 (3.6)	
	Severe	2 (0.2)	1 (0.2)	1 (0.2)	
	Extremely severe	1 (0.1)	1 (0.2)	0 (0.0)	
Anxiety (DASS)	Normal	946 (87.3)	465 (89.3)	481 (85.5)	0.098
	Mild	69 (6.4)	25 (4.8)	44 (7.8)	
	Moderate	40 (3.7)	16 (3.1)	24 (4.3)	
	Severe	27 (2.5)	15 (2.9)	12 (2.1)	
	Extremely severe	2 (0.2)	0 (0.0)	2 (0.4)	
Depression (DASS)	Normal	984 (90.8)	470 (90.2)	514 (91.3)	0.528
	Mild	57 (5.3)	32 (6.1)	25 (4.4)	
	Moderate	39 (3.6)	17 (3.3)	22 (3.9)	
	Severe	3 (0.3)	1 (0.2)	2 (0.4)	
	Extremely severe	1 (0.1)	1 (0.2)	0 (0.0)	

## Discussion

This study provides novel insights into the burden of Long COVID in a predominantly rural population of Northern India. To the best of our knowledge, it is the first to separately examine post-hospitalization and non-hospitalized patients while focusing on neurologic manifestations of Long COVID. We found that these two groups exhibit distinct demographic and clinical profiles. Post-hospitalization patients were older, less frequently vaccinated, had a higher BMI, and more often had a history of lung disease prior to COVID-19. These characteristics are consistent with known risk factors for severe COVID-19 pneumonia reported in studies from other countries (18, 19).

The frequency of neurologic symptoms was notably high, with more than two-thirds of participants reporting at least one neurologic manifestation approximately 2.5 years after disease onset. The most prevalent symptoms included myalgia (18.5%), dizziness (17.3%), headache (16.6%), pain (15.7%), anosmia (13.3%), and brain fog (9.4%), with only dizziness being more frequent among post-hospitalization patients. This contrasts with the relatively low prevalence of abnormal neurologic signs, present in only (7.6%), underscoring the high symptom burden despite largely unremarkable neurologic examinations. Similar long-term neurologic complaints have been documented in European and U. S. cohorts, supporting the hypothesis of persistent neuroinflammatory or dysautonomia mechanisms underlying Long COVID (20).

We also observed marked differences in general symptoms between the two groups. Non-hospitalized individuals were more frequently affected by fatigue, sleep problems, chest pain, and gastrointestinal symptoms, whereas post-hospitalization patients more often reported shortness of breath. These differences may reflect distinct underlying pathophysiologic mechanisms. In non-hospitalized individuals, persistent fatigue and GI disturbances may result from autonomic imbalance, low-grade inflammation, or post-viral immune dysregulation, as described in other international studies (21, 22). In contrast, dyspnea among post-hospitalization patients likely reflects residual pulmonary or cardiovascular sequelae following severe COVID-19 pneumonia.

Approximately 5% of all participants demonstrated objective cognitive impairment on a brief standardized cognitive test. This finding is concerning given the relatively young mean age of our cohort (<40 years), compared to the 10% prevalence of cognitive impairment reported in Indian urban elders (average age 70 years) (23). Educational disparities in our population, including over one-quarter of illiterate participants, may also influence cognitive testing performance. Longitudinal studies with larger, more diverse samples are needed to determine the long-term trajectory of cognitive dysfunction in Indian Long COVID patients.

Furthermore, around 10% of participants reported symptoms of stress, anxiety, and depression. Although these rates are higher than those reported in the general adult Indian population (2.6% for anxiety and 2.7% for depression), they are lower than rates reported from Western countries (21, 22, 32, 33). This difference may partly reflect cultural factors in India, where mental health issues remain stigmatized and may be underreported (24).

This study has limitations. Less than 6% of the patients contacted agreed to participate, possibly due to stigma associated with COVID-19 experienced by over one-third of recovered individuals in India (34), as well as logistical barriers such as travel distance and competing work responsibilities in rural areas, balanced with the perception of the importance of the symptoms. Nonetheless, our cohort remains representative of the Rajasthan population in terms of demographics, religion, and education.

Finally, data were collected an average of 2.5 years after symptom onset, which may introduce recall bias, a known limitation in long-term follow-up studies of infectious sequelae. Participants with more severe symptoms may have been more likely to respond, which could overestimate prevalence. Earlier evaluations following infection may have revealed an even greater symptom burden of Long COVID in the Indian population.

## Conclusion

There is nowadays more than 44 million COVID-19 survivors in India (2). By extrapolating the prevalence of Neurologic manifestations in our study to the entire country, it is expected that over 30 million people living in India have lingering neurologic symptoms of Long COVID 2.5 y later. However, this number is likely to increase, due to the higher risk of developing Long COVID after iterative episodes of COVID-19, despite vaccination and boosters (25, 26). Long COVID affects predominantly young and middle-aged individuals in their prime, causing enormous social and economic impact (12, 27).

Our results highlight the need for education about the occurrence of Long COVID in India, as well as improved screening and diagnosing of its neurologic manifestations. While the root causes of Long COVID remain to be fully identified (27–29), symptomatic treatment may alleviate many of its bothersome manifestations (30), and individuals suffering from cognitive dysfunction may benefit from targeted cognitive rehabilitation (31).

Long COVID, also called post-acute sequelae of SARS-CoV-2 infection (PASC) is defined as the persistence of symptoms 3 months after COVID-19 and had been recognized globally as a significant public health issue. Previous studies had documented the prevalence of Long COVID in India. However, research specifically focusing on neurological and psychiatric manifestations of Long COVID in the Indian population remained limited. There was also a lack of large-scale studies comparing post-hospitalized and non-hospitalized individuals in rural India, despite evidence suggesting differential symptom profiles based on acute disease severity.

This study is among the first in India to comprehensively compare neurocognitive and psychiatric symptoms between post-hospitalization and non-hospitalized Long COVID patients using standardized tools (MMSE and DASS-21). It highlights that nearly 70% of patients experience persistent neurological symptoms up to 2.5 years after infection. The study also shows a different symptom profile between post-hospitalization and non-hospitalized individuals. Importantly, objective cognitive impairment was detected in 5% of individuals with a mean age below 40, suggesting a concerning burden in a relatively young population. Additionally, psychological distress, though generally mild, was higher than national baseline levels.

The findings emphasize the need for long-term surveillance and rehabilitation programs tailored to both post-hospitalization and non-hospitalized COVID-19 survivors in India. Clinically, the results support the inclusion of routine cognitive and psychological screening in post-COVID follow-up, even for those with initially mild illness. For policy makers, the data call for investment in rural healthcare infrastructure, public awareness campaigns to reduce stigma, and incorporation of Long COVID management into national health guidelines. Future research may build on these results by exploring interventions to mitigate neurocognitive decline and improve quality of life for millions affected across the country.

## Data Availability

The raw data supporting the conclusions of this article will be made available by the authors, without undue reservation.
